# Applying Satellite‐Derived PM_2.5_ Data to Policy‐Relevant Air Quality Metrics

**DOI:** 10.1029/2025GH001585

**Published:** 2026-07-07

**Authors:** Tracey Holloway, Summer Acker, Lizzy Kysela, Colleen Heck, Aaron van Donkelaar, Randall V. Martin, Kevin M. Stewart, Katherine Pruitt

**Affiliations:** ^1^ Nelson Institute Center for Sustainability and the Global Environment University of Wisconsin—Madison Madison WI USA; ^2^ Department of Atmospheric and Oceanic Sciences University of Wisconsin—Madison Madison WI USA; ^3^ Department of Energy Environmental & Chemical Engineering Washington University in St. Louis St. Louis MO USA; ^4^ American Lung Association Chicago IL USA

**Keywords:** air pollution, geographic information systems (GIS), data fusion, clean air act, public health

## Abstract

Since 2000, the American Lung Association (ALA) has published an annual “State of the Air” report, which assigns grades and ranks United States (U.S.) cities and counties based on data from regulatory monitors reported to the Environmental Protection Agency (EPA). We evaluate satellite‐derived gridded data sets for fine particulate matter (PM_2.5_), as a possible method to support the ALA assessment in counties without regulatory monitors. Our analysis compares two publicly available, annual average, satellite‐derived gridded PM_2.5_ data sets, allocated to U.S. counties using three different methods. Assigning the 90th percentile gridded value of PM_2.5_ within a county as the county‐level indicator yields moderately strong agreement between ground‐based monitor PM_2.5_ results and satellite‐derived PM_2.5_ results, with a spatial correlation coefficient of 0.76 for 8 years of Washington University global (WashU GL) data. This agreement is evaluated with respect to concentration values, rankings, and the definition of counties as “passing” or “failing” the ALA annual PM_2.5_ benchmark of 9.0 μg/m^3^ (the 2024 EPA National Ambient Air Quality Standard for annual PM_2.5_). While most unmonitored counties are “passing” the ALA benchmark, we identify 63 counties that would be classified as “failing” if satellite‐based data were used to assign ALA grades. Improving methods to analyze satellite‐derived data for comparison with monitor‐based metrics can support broader utilization of space‐based data in the U.S. and globally.

## Plain Language Summary

Air pollution is a major public health concern, with fine particulate matter (PM_2.5_) linked to heart disease, lung disease, and premature death. The American Lung Association provides public‐facing data on air quality; however, data are currently limited to the ∼20% of counties with regulatory monitors for PM_2.5_. We compared methods of calculating county‐level PM_2.5_ from satellite‐derived data to best match county‐scale metrics from ground monitors. We found the best agreement by applying the 90th percentile to satellite values within each county. Applying this approach, we identified 63 unmonitored counties could be classified as having unhealthy and “failing” air if satellite data were used in grading. Our findings suggest that new methods can better align satellite data sets with decision‐relevant air quality metrics.

## Key Points

- A new approach is presented to compare gridded, satellite‐derived data and point‐based monitoring data at the U.S.‐county scale
- The 90th percentile‐grid in‐county method correlates best with EPA monitor PM_2.5_ data for ranking pollution levels across U.S. counties
- Satellite‐derived data extends air quality assessments to unmonitored U.S. counties, identifying areas that may exceed EPA PM_2.5_ standards

## 1 Introduction

Fine particulate matter (PM_2.5_) represents the leading environmental risk to health (Kloog et al., 2013; Yin et al., 2024). Exposure to ambient PM_2.5_ accounts for approximately 4 million premature deaths per year on a global basis (Cohen et al., 2017), and over 50,000 premature deaths in the United States (U.S.) (Health Effects Institute, 2024). In addition to premature mortality, exposure to PM_2.5_ can lead to respiratory and cardiovascular disease (Hoek et al., 2013; Peng et al., 2009), lung cancer (Li et al., 2018), neurological disorders (Fu et al., 2019; Li et al., 2022) and other diseases (Tian et al., 2017).

The term PM_2.5_ refers to liquids or solids suspended in the air with aerodynamic diameters less than 2.5 microns (μm), including both directly emitted particles (“primary” PM_2.5_) and chemically formed PM_2.5_ (“secondary” PM_2.5_). Primary PM_2.5_ can come from various sources, including open burning such as wildfires and agricultural fires, windblown dust, diesel fuel combustion, solid fuel combustion, and other sources (US EPA, 2016a). The chemical composition of PM_2.5_ varies by location, with mineral dust contributing more in the Southwestern U.S., while coastal cities experience higher levels of sea‐salt PM_2.5_ (van Donkelaar et al., 2019, 2021). Ammonium, nitrate, and sulfate are all types of secondary PM_2.5_ formed due to their precursors: ammonia (NH_3_), nitrogen oxides (NO_x_), and sulfur dioxide (SO_2_) emissions as well as volatile organic compounds (VOCs) (Heo et al., 2016). NH_3_ emissions come from agricultural sources (Ma et al., 2021; Wyer et al., 2022) and are most prominent in the central U.S. (Yu et al., 2018). NO_x_ emissions are highest in large cities (Goldberg, Lu, et al., 2019) due to emissions from on‐road and off‐road engines, along with other combustion sources (Tong et al., 2015), while coal‐fired power plants and industrial sources account for most SO_2_ emissions (Lin et al., 2018). Exposure to any of these compounds can vary depending on location, time, and season (Jin et al., 2019; van Donkelaar et al., 2019), and low‐income and historically disadvantaged communities experience higher levels of exposure (Gallagher & Holloway, 2022; Kelly et al., 2021).

To evaluate compliance with federal regulations, the U.S. Environmental Protection Agency (EPA) oversees a ground‐based monitoring network for PM_2.5_ and other pollutants. Carefully calibrated and quality controlled, these monitors are considered the “gold standard” for measuring exposure relevant to human health (Diao et al., 2019; Grainger & Schreiber, 2019; Miranda et al., 2011). The placement and location of these ground‐based monitors are determined based on criteria set by the EPA and include densely populated areas, near large stationary sources of pollution, and high‐density traffic locations (US EPA, 2008). Measurements from these air quality monitors are reported through the EPA Air Quality System (AQS) and used to evaluate compliance with the National Ambient Air Quality Standards (NAAQS), as well as tracking air quality trends, informing regulatory and research activities, and supporting public‐facing air quality outreach.

Perhaps the highest profile air quality outreach effort in the U.S. is the annual “State of the Air” (SOTA) report from the American Lung Association (ALA), first published in 2000 (ALA, 2025b). In recent years, the ALA has also released two supplemental reports in the “Something in the Air” series—one focusing on satellite‐derived PM_2.5_ concentrations in unmonitored counties (ALA, 2024), and another highlighting nitrogen dioxide (NO_2_) exposure disparities and monitoring limitations (ALA, 2025a). The ALA's SOTA report acts as an air quality “report card,” assigning grades using the EPA AQS data, and it ranks U.S. cities and counties based on ozone concentrations and 24‐hr and annual PM_2.5_ concentrations. For annual PM_2.5_, the ALA uses EPA county‐level design values (DVs), which are calculated by the EPA as the four‐quarter average of quarterly averaged measurements of PM_2.5_ taken at regulatory monitors and averaged over the preceding 3 years (e.g., 2016, 2017, and 2018 data is used to calculate the 2018 DV which is made available in 2019). The DVs for each monitor are calculated by the EPA using the Federal Reference Method or equivalent data reported by state, tribal, and local monitoring agencies.

In determining county‐scale rankings or pass‐fail grades, the ALA utilizes the maximum, monitor‐scale, annual PM_2.5_ DV provided by the EPA, where the maximum value in each county is assigned to the county, hereafter referred to as the county DV (CDV). The ALA uses these CDVs to assign grades to counties, with a passing grade for counties meeting the EPA annual PM_2.5_ standard and a failing grade for those exceeding it. Throughout the 2023 SOTA report, the ALA applied the 2012 NAAQS criteria for annual PM_2.5_, defining passing as CDV ≤ 12.0 μg per cubic meter (μg/m^3^) and failing as CDV ≥ 12.1 μg/m^3^. The ALA assigns a county an “incomplete” grade if there is insufficient data to determine the CDV (ALA, 2025b). Starting with the 2024 SOTA report, the ALA adopted the revised 2024 NAAQS criteria for annual PM_2.5_ such that passing is defined as CDV ≤ 9.0 μg/m^3^ or failing as CDV ≥ 9.1 μg/m^3^.

Here we evaluate the potential for satellite‐derived PM_2.5_ to support the pass/fail grading and rankings for annual PM_2.5_, with the goal of informing air quality levels in unmonitored counties. Most U.S. counties lack a monitor, especially less populated regions (Di et al., 2019; Goldberg, Gupta, et al., 2019) where nonurban emissions from wildfires or agricultural sources can produce high levels of atmospheric PM_2.5_ (McDuffie et al., 2021). Even within a monitored county, point‐based monitor measurements may not provide a full representation of air quality patterns (Diao et al., 2019). Advanced methods have been developed to fuse satellite retrievals of aerosol optical depth (AOD), atmospheric models, and ground‐measurements to calculate near‐surface PM_2.5_ (Holloway et al., 2025). Satellite instruments do not directly measure PM_2.5_ concentrations but rather are applied to retrieve AOD, a unitless measure of light extinction due to particles in a column of air above the earth's surface (van Donkelaar et al., 2010). Various retrieval methods applied to data from multiple space‐based instruments have been used to calculate AOD (Hsu et al., 2013, 2019; Lyapustin et al., 2018; Martonchik et al., 2009; Sawyer et al., 2020; Sayer et al., 2012, 2013), which have been combined with models and ground‐based measurements to estimate PM_2.5_ (Holloway et al., 2021) with promising accuracy (Martin et al., 2019; Shaddick et al., 2018; van Donkelaar et al., 2016). While performance depends on methodology and validation techniques, hybrid geophysical‐statistical methods that combine satellite AOD retrievals, chemical transport model outputs, and ground‐based monitoring data have improved the precision of PM_2_._5_ estimation, with *R* ^2^ ranging from ∼0.79 to 0.84 (Beckerman et al., 2013; Di et al., 2016; van Donkelaar et al., 2016).

Evaluation of gridded, satellite‐derived data sets have primarily been conducted on a grid‐to‐monitor or area‐averaged basis. However, this approach differs conceptually from the CDV approach discussed above, wherein the highest monitor in a county is used to determine the evaluation metrics. Using two publicly available satellite‐derived PM_2.5_ data sets representing years 2016 or later, we calculate a county design value equivalent (CDVE) for each county using alternate approaches and compare it to the CDV calculated based on monitoring data. Using the best‐performing CDVE method, we provide a proof of concept for integrating satellite‐derived data into pass/fail categorizations and rankings for all U.S. counties.

This study evaluates agreement between estimates of PM_2.5_ from data fusion products and ground‐based monitors, with the goal of broadening the utilization of satellite‐derived PM_2.5_ in health, public engagement, and regulatory contexts. This work also highlights the need for a method to adjust the satellite‐derived CDVEs to better match the CDVs, and the value of comparing findings across data sets as they improve with advanced methods like machine learning, and advancing data inputs (models, satellites, and ground‐based data).

## 2 Materials and Methods

Data fusion estimates of near‐surface PM_2.5_ offer spatially contiguous near‐surface PM_2.5_ using an atmospheric chemistry model and/or ground observations along with AOD from one or more space‐based instruments. We collected and evaluated all such recent (2016–2023) publicly available data fusion PM_2.5_ products over the continental U.S. Table S1 in Supporting Information S1 provides a complete summary of each data set compilation and spatiotemporal resolution, which use different models, satellites, and/or algorithms. Resolution of available data sets varied from 1 km × 1–75 km × 75 km, including both gridded and geographic allocations (e.g., county, census tract, varying in size).

Publicly available, gridded data sets with resolution finer than 12 km × 12 km with data extending beyond 2016 were further evaluated in this study. These criteria narrowed the data options to two products (Table S1 in Supporting Information S1): (a) The WashU North American (NA) Regional Estimates V5. NA.04 (van Donkelaar et al., 2024) and (b) The WashU Global/Regional (GL) Estimates V5. GL.05 (van Donkelaar et al., 2021). Both data sets are based on AOD from multiple satellite instruments (MODIS/Terra, MODIS/Aqua, MISR/Terra, SeaWiFS/SeaStar, VIIRS/SNPP, and VIIRS/NOAA20) using their respective retrievals (Dark Target, Deep Blue, MAIAC, MISR) that are combined and related to PM_2.5_ using the GEOS‐Chem chemical transport model, followed by a subsequent statistical fusion that incorporates additional information from PM_2.5_ measurements (van Donkelaar et al., 2021, 2024). Although algorithmically similar to WashU GL, WashU NA makes use of a more recent GEOS‐Chem version in its High‐Performance configuration (v13.2.1) with daily biomass burning emissions and includes additional predictor variables during its statistical fusion. Both data sets cover the contiguous U.S. on a 0.01° × 0.01° grid. WashU NA and GL data sets cover all of Alaska and Hawaii as well, except for one county in Alaska (North Slope). The WashU GL data product additionally quantifies annual mean uncertainty, which is based on the quality of the AOD retrievals and their relationship with PM_2.5_ (van Donkelaar et al., 2021). Bright surfaces are known to incur errors in AOD and associated PM_2.5_ estimates, as seen over the White Sands Missile Range in southern New Mexico, Great Salt Lake in Utah, and other desert areas. We used the WashU GL uncertainty estimate to remove values with greater than 90% uncertainty, and pixels that were removed from the GL data set were also removed from the NA data set. To minimize the potential for negative impact of frequently high uncertainty locations on the county‐wide representation in the NA data set, pixels (grids) that exceeded the 90% uncertainty threshold for more than 25% of years were removed from the entire times series prior to averaging by period.

Satellite‐derived data were used for the years 2016 through 2023, all of which have been attributed to the U.S. Census Bureau 2020 county boundaries shapefile (US Census Bureau, 2025). There have been boundary and status changes to some counties between 2015 and 2020, although none of these counties had an EPA monitor. Therefore, the use of 2020 county boundaries does not affect study results.

Monitor‐based county‐level DVs were accessed from the EPA “Air Quality Design Values” page (US EPA, 2016b) for the 2016–2018 DV period (2018 DV) through the 2021–2023 DV period (2023 DV). Python GeoPandas was used to assign the mean monitored DV to each county to calculate CDVs, and to assign 90th‐percentile, mean, and maximum gridded satellite‐derived data to each county to calculate the CDVEs.

Pearson's correlation coefficient (*r*, Equation 1) was calculated between the CDVs and CDVEs to determine the strength and relation between the linear relationship (i.e., variables move in the same direction at a constant rate), and Spearman's rank correlation (*r*_s,_ Equation 2) was calculated to determine the strength and direction of monotonic relationships (i.e., variables move in the same direction but not necessarily at a constant rate).

(1) $$\text{Pearso}n’s\;\text{Correlation}\;\text{Coefficent}\;\left(r\right)=\frac{\sum \left(\text{CDV}-\bar{\text{CDV}}\right)\left(\text{CDVE}-\bar{\text{CDVE}}\right)}{\sqrt{\sum {\left(\text{CDV}-\bar{\text{CDV}}\right)}^{2}\sum {\left(\text{CDVE}-\bar{\text{CDVE}}\right)}^{2}}}$$

(2) $$\text{Spearma}n’s\;\text{Rank}\;\text{Correlation}\;\text{Coefficent}\;\left(r_{s}\right)=1-\frac{6\sum d_{i}^{2}}{n\left(n^{2}-1\right)}$$

In Equation 1, the CDV is the county maximum monitor‐based DV for a given period, $\bar{\text{CDV}}$ is the spatial mean of the values, CDVE is the calculated satellite‐derived CDVE at those same locations (using 90th‐percentile, mean, and maximum grid approaches), and $\bar{\text{CDVE}}$ is the spatial mean of those values. In Equation 2, *d*_i_ is the difference between the ALA rank based on CDV and the calculated rank based on alternate approaches to calculated CDVE; *n* is the number of counties ranked.

## 3 Results

To compare methodologies of CDVE calculation, we consider calculations based on a (a) 90th‐percentile threshold; (b) area average (mean‐grid); and (c) maximum grid in the county (max‐grid). The 90th‐percentile threshold was determined based on a calculation of CDVEs for all percentiles from the 0.01 to the 0.99 of the WashU GL PM_2.5_ estimates (van Donkelaar et al., 2021) for multi‐year periods (2016–2018 through 2021–2023), as well as calculated Pearson and Spearman correlations between these CDVEs and CDVs (for best percentiles of each DV period see Table S2 in Supporting Information S1; for 60th–95th percentiles see Figure S1 in Supporting Information S1). Across the time periods, optimal percentiles ranged from 0.75 to 0.98 for Pearson and 0.93 to 0.98 for Spearman. Averaging the optimal percentiles across year ranges yielded an overall best percentile of 0.88 for the Pearson correlation and 0.95 for the Spearman rank correlation, which together average to 0.92. A parallel analysis using the WashU NA PM_2.5_ estimates (van Donkelaar et al., 2024) data set produced similar results, with a best overall Pearson percentile of 0.86, a best overall Spearman percentile of 0.96, and a combined average of 0.91. To reflect these results, recognizing uncertainties and year‐to‐year variability in the calculations, we use the 90th percentile for comparison with the mean‐grid and max‐grid method.

**Figure 1:**
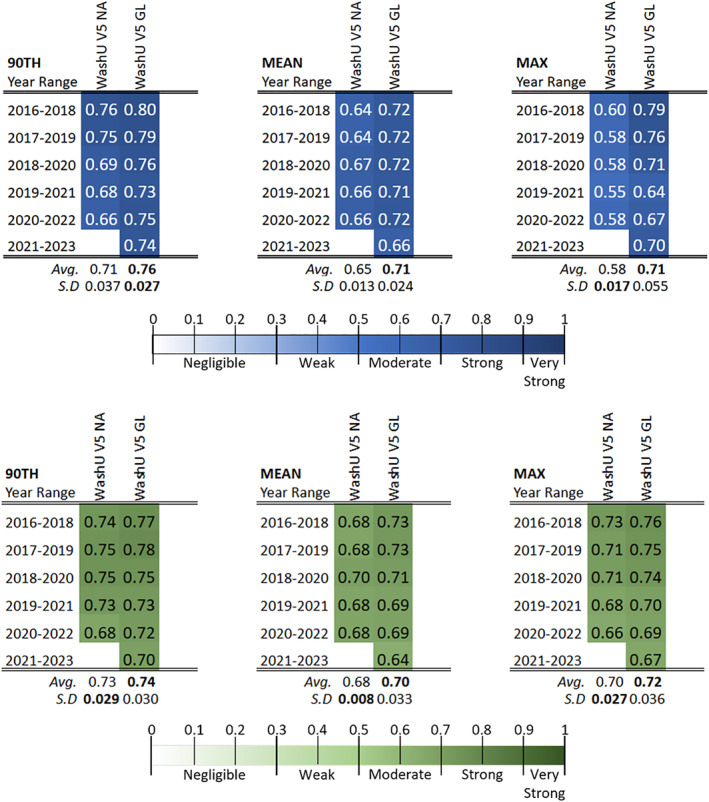
(Top Row) The 2016–2023 Pearson Correlations, above in blue, for the WashU NA and WashU GL data sets using the 90th percentile, mean, and max methods; (Bottom Row) The 2016–2023 Spearman Rank Correlations, below in green, for the WashU NA and WashU GL data sets using the 90th percentile, mean, and max methods.

Figure 1 (top row) shows the Pearson Correlation coefficients (*r*) for the 3‐year averages for 2016 through 2023, comparing 90th percentile‐grid, mean‐grid and max‐grid CDVE calculation methods. At the time of our study (2022–2025), WashU NA extended through 2022; and WashU GL through 2023. Highest agreement between CDVs and CDVEs are found using the 90th percentile method (90th; left panel). Applying the 90th percentile grid captures peak values within a county, in a manner akin to CDV and max‐grid but can reduce the impact of extreme values in the two satellite‐derived data sets. Using this approach, the average correlation coefficient $\underline{r}$_grid90th_ of 0.71 was found for WashU NA (for 7 years' data) and 0.76 was found for WashU GL (for 8 years' data). These correlation coefficients suggest a moderately strong level of agreement between monitor‐based CDVs and all monitored CDVEs using the 90th percentile method.

Calculated *r* values for the maximum (average NA *r* = 0.58; average GL *r* = 0.71) and mean (average NA *r* = 0.65; average GL *r* = 0.71) methods also show moderate to strong correlations across all three data sets. The WashU GL data exhibits somewhat higher *r* values than WashU NA values for all methods. We expect this is due to the WashU NA data set having greater extreme values than the WashU GL data set, even after additional filtering was applied to the NA data set (as discussed in Materials and Methods).

A similar pattern is found for the agreement in rankings based on Spearman Rank Correlation, shown in Figure 1(bottom row). Unlike the Pearson Correlation coefficient, which assumes a linear relationship, the Spearman Rank correlation provides information about the strength and direction of a monotonic relationship and allows comparison of the county ranking as calculated by the CDVs and CDVEs. As with overall spatial correlation, the 90th percentile method shows the strongest relationship (average *r*_s_ NA = 0.73; average *r*_s_ GL = 0.74), followed by the max method (average *r*_s_ NA = 0.70; average *r*_s_ GL = 0.72), then the mean method (average *r*_s_ NA = 0.68; average *r*_s_ GL = 0.70). We find moderate consistency between satellite‐derived CDVEs and ground‐based CDVs, ranging from *r* ≈ 0.68 to 0.74 depending on the data set and aggregation method.

Because the monitor‐based CDVs are based on the maximum monitored value with a county, conceptually the maximum‐grid approach is the best analog for gridded data. However, the maximum‐grid CDVE is sensitive to outliers, errors, or localized extreme events (e.g., wildfires), in a way that the monitor‐based CDV is not, due to ongoing quality control, guidelines on monitor placement, and flagging Exceptional Events for exclusion from the data set. Given these differences between monitor and satellite‐derived data, the 90th percentile approach best represents an analog for CDV based on the satellite‐derived CDVE.

### 3.1 County‐Level “Pass” and “Fail” Grades

Using the 90th‐percentile CDVE methodology, and the WashU GL 2023 CDVE values, passing and failing grades are calculated for all U.S. counties in a manner comparable to the ALA SOTA report with a 12.0 μg/m^3^ threshold as well as the updated 9.0 μg/m^3^ threshold. Nearly 100% of counties have a CDVE: 3,142 out of 3,143 total U.S. counties, all except one county in northern Alaska with inadequate data coverage. Monitor‐based CDVs were calculated for 17.1% of U.S. counties, with 536 counties having at least one monitor reporting an EPA 2023 DV.

Comparing the 536 counties with both CDV and CDVE, we find that the CDVE has a positive, mean bias compared to CDV values of 0.07 μg/m^3^ (i.e., the mean bias error) with differences ranging from −5.33 to 5.73 μg/m^3^, a mean absolute difference of 0.90 μg/m^3^ (i.e., the mean absolute error), a root mean square error of 1.23 μg/m^3^,  and an absolute difference standard deviation of 0.84 μg/m^3^.

**Figure 2:**
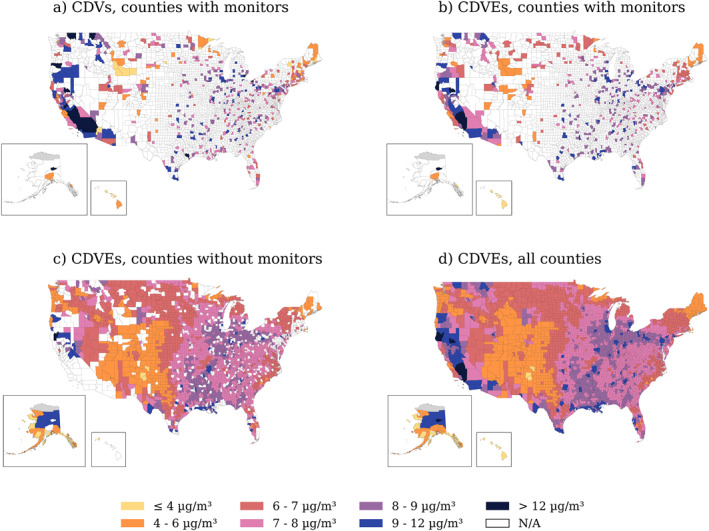
(a) CDVs in counties with monitors, calculated from monitor‐based measurements; (b) CDVEs in counties with monitors (the same counties shown in panel (a)), calculated from the 90th‐percentile WashU GL 2023 CDVEs; (c) CDVEs in counties without monitors; and (d) CDVEs across all counties. All counties are shown except North Slope Borough, Alaska (gray). Bins are defined such that each range excludes the lower bound and includes the upper bound (e.g., 6 < *x* ≤ 7 μg/m^3^ is labeled as 6–7 μg/m^3^), with the exception of the first bin (≤4 μg/m^3^) and the last bin (>12 μg/m^3^).

Figure 2 presents spatial distributions of the 2023 monitor‐based CDVs and the 90th percentile WashU GL 2023 CDVEs across U.S. counties, with separate panels showing: (a) CDVs, counties with monitors; (b) CDVEs, counties with monitors; (c) CDVEs, counties without monitors; and (d) CDVEs, all counties. These panels show where counties approach or exceed the EPA PM_2.5_ standards. Table 1 provides a detailed breakdown of the county‐level 90th percentile CDVEs seen in Figure 2, categorizing counties into ranges relative to the EPA's PM_2.5_ standards.

**Table 1:** The County‐Level 90th Percentile WashU GL 2023 CDVEs (Blue) and 2023 Monitor‐Based CDVs (Orange), Grouped by Compliance Thresholds Relative to the 2012 and 2024 NAAQS for Annual PM_2.5_

PM_2.__5_ range (μg/m^3^)	(#) counties (CDV)	(#) Counties (CDVE)	(%) Total counties	(#) Counties with monitors	(#) Counties without monitors	(%) Counties without monitors
***x* > 12.0**	**16**	**8**	**0.3%**	**7**	**1**	**12.5%**
**9.0 < *x* ≤ 12.0**	**99**	**163**	**5.2%**	**101**	**62**	**38.0%**
8.0 < *x* ≤ 9.0	138	683	21.7%	182	501	73.4%
7.0 < *x* ≤ 8.0	149	1,208	38.4%	121	1,087	90.0%
6.0 < *x* ≤ 7.0	67	737	23.5%	85	652	88.5%
4.0 < *x* ≤ 6.0	54	323	10.3%	36	287	88.9%
*x* ≤ 4.0	13	20	0.6%	4	16	80.0%
Total	536	3,142	100%	536	2,606	82.9%

*Note.* In the “PM_2.__5_ Range” column, *x* represents the county‐level value: CDV for monitor‐based values and CDVE for satellite‐derived values. The table shows the number and percentage of counties in each PM_2.__5_ range, separated into counties with and without monitors, along with the percentage of counties without monitors. The bold color in this table refers to value ranges that have been used to define “failing” grades. The orange is used to distinguish the monitor‐based CDVs from the satellite‐based CDVEs and the CDVE county statistics which are shown in blue.

The monitor‐based CDV method results in a higher number of “failing” counties, both against the 2012 NAAQS value of 12.0 μg/m^3^ threshold and the 2024 NAAQS 9.0 μg/m^3^ threshold (Table 1). Relative to the 2012 NAAQS and among counties with monitors, the CDV method finds 16 counties (3.0%) above 12.0 μg/m^3^; the satellite‐derived CDVE finds 7 counties (1.3%). Relative to the 2024 NAAQS and among counties with monitors, the CDV method finds 115 counties (21.5%) above 9.0 μg/m^3^; the satellite‐derived CDVE, 108 counties (20.1%). There are 63 unmonitored counties that are identified by the CDVE method to be graded as “failing” relative to the 2024 NAAQS value. These findings indicate that the 90th percentile method with satellite‐derived CDVEs yields more conservative results than the monitor‐based CDV method, but that the two methods are not always identifying the same counties (discussed further in Acker et al., 2026).

Differences in spatial sampling between satellite‐derived surface PM_2.__5_ concentration estimates and ground‐based PM_2.__5_ measurements impact the agreement between CDV and CDVE. While satellite‐derived PM_2.5_ offers greatly increased spatial sampling compared to ground‐based monitors, additional uncertainties can be present in satellite‐derived PM_2.__5_ estimates. Satellite‐derived PM_2.__5_ estimates are based on total column AOD retrievals, with uncertainties arising from both the AOD retrieval itself and in its conversion into surface PM_2.__5_ estimates, as this conversion relies on an accurate representation of aerosol vertical profiles, chemical composition, and relative humidity effects (van Donkelaar et al., 2010, 2016, 2019). Satellite AOD retrievals are not possible in all conditions and are aggregated from available daily values to monthly periods. Although efforts are made to adjust for sampling effects, data aggregation can introduce biases, especially in regions with strong seasonal variability or extreme pollution events (Levy et al., 2009; Paciorek & Liu, 2009; van Donkelaar et al., 2010). Smoke plumes from wildfires can bias satellite AOD estimates, generally resulting in the underestimation of surface PM_2.__5_ concentrations during such events (Geng et al., 2018; Ye et al., 2022). Even after statistical calibration with ground‐based monitors, regional biases can remain in areas with sparse monitoring (van Donkelaar et al., 2016, 2019). Acker et al. (2026) presents a detailed analysis of the county characteristics associated with higher‐ or lower‐level of agreements between CDV and CDVE values calculated with the 90th percentile methodology discussed here.

Of the 536 counties with monitors, the monitor‐based CDV method identifies 16 counties (2.9%) with CDVs above 12.0 μg/m^3^, whereas the satellite‐derived CDVE method identifies 7 counties. Indeed, we expect a higher percentage of monitored counties to show failing values because monitors are often located in counties at risk of high PM_2.5_ levels. The CDV method identifies 99 counties (18.5%) with a CDV greater than 9.0 μg/m^3^ and less than or equal to 12.0 μg/m^3^; in these moditored counties, 101 have CDVEs greater than 9.0 μg/m^3^ and less than or equal to 12.0 μg/m^3^. Counties with CDVEs less than or equal to 9.0 μg/m^3^ account for 2,971 counties (94.6%), mostly unmonitored.

Of the 2,606 counties without monitors evaluated using the CDVE, 1 (0.04%) would be graded failing against a 12.0 μg/m^3^ threshold: a county in northern California, where the high values were likely affected by wildfires from 2021 to 2023. Of these same counties without monitors, 63 (2.4%) would be graded failing against a 9.0 μg/m^3^ threshold, spread across 19 states (Figure 2). These 63 unmonitored counties align with known regions of elevated PM_2.5_ air pollution, for example, areas affected by industrial and transportation sources in California's valley region (Hasheminassab et al., 2014) and petrochemical activity in the Gulf Coast region (Russell et al., 2004). Acker et al. (2026) evaluates the likelihood of CDVEs and CDVs aligning on county classification of air pollution status in all U.S. counties, finding that monitoring characteristics (especially low monitor count) and geographical characteristics including low urbanization, county size, mountains, deserts, and wildfires increase the likelihood of discrepancy between CDV and CDVE values.

### 3.2 County‐Level Pollution Rankings

**Figure 3:**
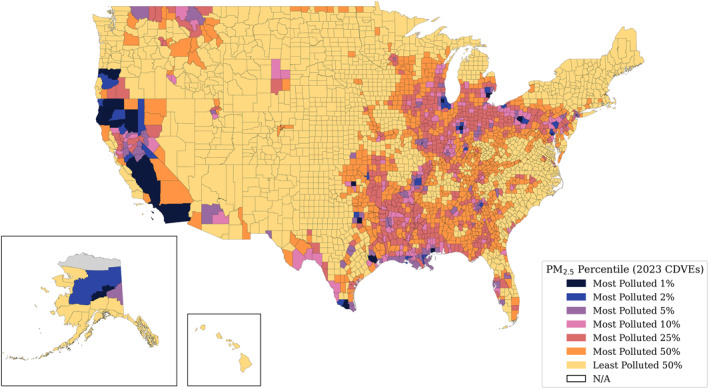
A spatially contiguous version of the rankings for all counties in the U.S. based on the WashU GL 2023 CDVEs. All but one county (shown in gray in Alaska) was ranked.

Figure 3 shows all CDVE counties ranked based on the WashU GL 2023 CDVE 90th‐percentile method to illustrate the geographic distribution of annual average PM_2.5_ pollution in U.S. counties (also reportd in Table 2). CDVEs greater than or equal to 10.43 μg/m^3^ represent the counties ranked as the most polluted 1% (99th percentile); 9.77–10.43 μg/m^3^, the most polluted 2% (98th to 99th percentile); 9.10–9.77 μg/m^3^, the most polluted 5% (95th to 98th percentile); 8.60–9.10 μg/m^3^, the most polluted 10% (90th to 95th percentile); 8.10–8.60 μg/m^3^, the most polluted 25% (75th to 90th percentile); 7.43–8.10 μg/m^3^, the most polluted 50% (50th to 75th percentile); and less than 7.43 μg/m^3^, the least polluted 50% (less than 50th percentile). These rankings reflect multi‐year averages that reduce the influence of episodic events but still reveal counties with the highest pollution exposure.

**Table 2:** County‐Level Pollution Rankings Defined Using Satellite‐Derived 90th‐Percentile WashU GL 2023 CDVEs, With 2023 Monitor‐Based CDVs

(%) Most polluted	PM_2.__5_ range (μg/m^3^)	(#) Counties (CDV)	(#) Counties (CDVE)	Rank range	Percentile (perc.) range	(#) Counties with monitors	(#) Counties without monitors	(%) Counties without monitors
1%	*x* ≥ 10.43	31	31	1 to 31	*x* ≥ 99th perc.	26	5	16.1%
2%	9.77 ≤ *x* < 10.43	30	31	32 to 62	98th perc. ≤ *x* < 99th perc.	17	14	45.2%
5%	9.10 ≤ *x* < 9.77	54	95	63 to 157	95th perc. ≤ *x* < 98th perc.	56	39	41.1%
10%	8.60 ≤ *x* < 9.10	67	157	158 to 314	90th perc. ≤ *x* < 95th perc.	67	90	57.3%
25%	8.10 ≤ *x* < 8.60	71	471	315 to 785	75th perc. ≤ *x* < 90th perc.	114	357	75.8%
50%	7.43 ≤ *x* < 8.10	91	786	786 to 1,571	50th perc. ≤ *x* < 75th perc.	91	695	88.4%
Lower 50%	*x* < 7.43	192	1,571	1,572 to 3,142	*x* < 50th perc.	165	1,406	89.4%
	Total	536	3,142	3,142	3,142	536	2,606	82.9%

*Note.* In the “PM_2.__5_ Range” column, *x* represents the county‐level value (CDV for monitor‐based counties and CDVE for satellite‐derived counties). The CDV column (orange) reports the number of monitored counties whose CDVs fall within each PM_2.__5_ concentration range. CDVE‐related columns (blue) report, for each pollution category (e.g., top 1%, top 2%, etc.), the total number of counties, rank range, percentile range, number of counties with monitors, number of counties without monitors, and the percentage of counties without monitors. Rank ranges use a split‐tie approach: when multiple counties share the same CDVE, the tied group is assigned ranks evenly above and below the tie boundary. The orange is used to distinguish the monitor‐based CDVs from the satellite‐based CDVEs and the CDVE county statistics which are shown in blue.

Among these counties, 5 counties in the most polluted 1% lack monitors (in California, Alaska and Maryland) and 14 counties in the most polluted 2% lack monitors, encompassing a mix of rural and urban regions. In the most polluted 5%, 39 lack monitors and 90 counties in the most polluted 10% lack monitors. More than half of the counties in the top 10% most polluted category remain unmonitored. Among the least polluted 50%, most counties are unmonitored (89.4%).

California has the most counties with CDVEs in the most polluted 1%. CDVEs in the most polluted 2%–5% extend north from California through Oregon, likely associated with wildfire smoke, industry, and transportation (Carreras‐Sospedra et al., 2024; Meng et al., 2019); along the southern border of the U.S. from California to Florida, likely associated with natural mineral dust, biogenic secondary organic aerosol, high traffic and unpaved roads from the Mexican border (Meng et al., 2019; Rubio et al., 2022; US EPA, 2024), as well as industrial activities (US EPA, 2024); and much of the central U.S.

## 4 Conclusions

The ALA's SOTA report offers a high‐impact context to consider satellite‐derived data relevance to public‐facing, air quality information. The report presents county‐scale grades and rankings, based on the EPA‐calculated DVs at regulatory monitors, for three air pollution metrics: ozone (based on maximum daily 8‐hr averages), short‐term PM_2.5_ (based on daily averages), and long‐term PM_2.5_ (based on annual averages). Of these, long‐term PM_2.5_ is best‐suited for analysis with satellite‐derived data fusion products, with a growing number of data sets, using various fusion approaches and component data, developed for annual PM_2.5_ estimation (Kelly et al., 2021). Although the ALA uses EPA regulatory monitoring data to assign grades, these differ from regulatory attainment designations for passing or failing the NAAQS, which also considers Exceptional Events, potential partial county designations, or other factors impacting compliance with the Clean Air Act.

We present an analysis method to calculate county‐scale grades and rankings for annual average PM_2.5_ with satellite‐derived data. We find that calculating county‐scale values based on the 90th percentile of gridded values captures high pollution levels while modulating sensitivity to grid values with potential errors. Only the Global and North American data fusion products from WashU offer publicly available data for years more recent than 2017 across the U.S. at a 0.01° × 0.01° resolution. Since the WashU GL data set provides higher consistency with EPA‐calculated CDVs, most of our analysis uses this data set.

We note that our findings do not imply a validation of the satellite‐derived data sets. Rather, the observed agreement reflects the ability of satellite‐derived measurements to capture data that can be used to generate county‐scale estimates comparable to values obtained from ground‐based monitors. In particular, the ability of the highest grids of satellite‐derived data in a county to capture the values of the highest monitor in a county. Differences between CDVs and CDVEs may arise not only from limitations in satellite‐derived data but also from the spatial distribution of monitors, which may not capture the actual highest PM_2.5_ levels in a county. The potential mismatch of monitor location and maximum surface concentration may have increased in recent years, as wildfire activity has led to more extreme PM_2.5_ concentrations in areas that are less monitored. As a result, ground‐based monitors may be becoming less representative of the most polluted locations, which contributes to discrepancies between satellite‐ and monitor‐based estimates.

Despite these considerations, the 90th percentile method offers a practical approach for aligning satellite‐derived estimates with county‐scale monitor values. When allocating the 90th percentile grid value of PM_2.5_ in a county, the WashU GL product shows moderately strong spatial agreement with values calculated from the highest monitor value in the county (average *r* = 0.76 from 2016 to 2023). This product also shows moderately strong agreement with the county‐scale rankings calculated from the highest monitor value in the county (average *r*_s_ = 0.74 from 2016 to 2021).

Overall, the satellite‐based CDVEs appear to be more conservative than monitor‐based CDVs, in that fewer counties would be graded as “failing” when evalated with satellite‐derived data. Inconsistent identification as to which counties pass and fail with each approach has been analyzed by Acker et al. (2026). Reconciling these discrepancies should inform next‐generation data products for satellite‐derived PM_2.5_. For example, new satellite‐derived data sets could support the CDVE application, with model algorithms and data weighting focused on capturing peak values relevant to policy applications.

The value of satellite‐derived data to complement monitor‐based metrics, at their current level of agreement, will depend on the priorities and constraints of health and air quality organizations. For example, spatial agreement between CDVs and CDVEs may be compared with CDVs a calculated by the EPA Community Multiscale Air Quality model (US EPA, 2022).

This study highlights the potential for satellite‐derived data to inform the policy‐relevant design value metric, which is currently based on monitoring data. Hypothetical metrics could be designed for use with satellite‐derived data, for example for NAAQS evaluation as presented in Sullivan and Krupnick (2018). Other approaches from the literature have included the calculation of high‐resolution PM_2.5_ accounting for land use characteristics (e.g., Lu et al., 2025), and consideration of population‐weighted PM_2.5_ rather than area‐weighted concentrations (e.g., Li et al., 2017). Despite these potential new frameworks, ground‐based monitors remain the backbone of regulatory air quality assessment in the U.S. As a result, the uptake of satellite‐derived PM_2.5_ by U.S. air quality managers and related health professionals has been limited (Holloway et al., 2025).

Here, we focus on the potential for satellite data to complement, rather than replace, existing ground‐based monitors while also highlighting the need to establish additional ground‐based monitors. We explore the potential role of satellite‐derived data within current U.S. air quality decision‐making frameworks. Further advances in satellite technology, data product development, and successful applications hold the potential to broaden air quality management in a cost‐effective, inclusive manner, with particular value to rural areas, large states, and counties with limited monitors.

## Conflict of Interest

The authors declare no conflicts of interest relevant to this study.

## Supporting information

Supporting Information S1

## Data Availability

All data used in this study are open to the public. All Washington University Global and North American data was downloaded from the Washington University in St. Louis Atmospheric Composition Analysis Group (https://sites.wustl.edu/acag/datasets/surface‐pm2‐5/). The EPA county‐level design value data was downloaded from the EPA Air Quality Design Values page (https://www.epa.gov/air‐trends/air‐quality‐design‐values). The U.S. 2020 county shapefiles were downloaded from the U.S. Census Bureau Cartographic Boundary Files page (https://www.census.gov/geographies/mapping‐files/time‐series/geo/cartographic‐boundary.2020.html#list‐tab‐1883739534). All analysis code for this study is available in Zenodo (sjacker2, 2025; https://doi.org/10.5281/zenodo.17584000).
