# Supplementary material for: Applying Satellite‐Derived PM2.5 Data to Policy‐Relevant Air Quality Metrics
Source: Geohealth. 2026 Jul 7;10(7):e2025GH001585. doi: 10.1029/2025GH001585 (PMC13339721; doi:10.1029/2025GH001585)
Supplement: Supplementary file 1 — Supporting Information S1 [file GH2-10-e2025GH001585-s001.docx]

*GeoHealth*

Supporting Information for

Applying Satellite-Derived PM_2.5_ Data to Policy-Relevant Air Quality Metrics

Tracey Holloway^1,2*^, Summer Acker^1^, Lizzy Kysela^1,2^, Colleen Heck^1,2^, Aaron van Donkelaar^3^, Randall V. Martin^3^, Kevin Stewart^4^, Katherine Pruitt^4^

^1^Nelson Institute Center for Sustainability and the Global Environment, University of Wisconsin—Madison; Madison, 53705, USA.

^2^ Department of Atmospheric and Oceanic Sciences, University of Wisconsin—Madison; Madison, 53705, USA.

^3^ Department of Energy, Environmental & Chemical Engineering, Washington University at St. Louis; St. Louis, 63130, USA.

^4^ American Lung Association, Chicago, 60601, USA.

**Contents of this file**

Tables S1 to S2

Figure S1

**Additional Supporting Information**

Captions for Tables S1 to S2; Figure S1

References for Tables S1 to S2; Figure S1

**Supporting Information**

Table S1. All recent (2016-2025) publicly available data fusion fine particulate matter (PM_2.5_) products over the continental United States (U.S.)

| Dataset | Data Source | Temporal Coverage and Resolution | Spatial Resolution | | | | | | | | | Remote Sensing AOD and Retrieval Method | | | | | | | | Ground-Based Data | | | | | | | Reference | |
| --- | --- | --- | --- | --- | --- | --- | --- | --- | --- | --- | --- | --- | --- | --- | --- | --- | --- | --- | --- | --- | --- | --- | --- | --- | --- | --- | --- | --- |
|  |  |  |  |  |  |  |  |  |  |  |  | MODIS: Terra/Aqua | | | MISR | Sea-WiFS | VIIRS: SNPP/NOAA | | |  |  |  |  |  |  |  |  |  |
|  |  |  | 0.01° x 0.01° | 1 x 1 km^2^ | 3 x 3 km^2^ | National | State | Country | Zipcode | Census Tract | Block Group | DB | DT | MAIAC | MISR | DB | DB | DT | MAIAC | U.S. EPA AQS | AirNow | OpenAQ | SPARTAN | WHO | GBD | Other |  |  |
| Global Estimates V6.GL.02.04 | WashU | 1998-2023 Annual & Monthly | x |  | x | x |  |  |  |  |  | x | x | x | x | x | x | x |  | x |  | x | x | x | x | x | (Atmospheric Composition Analysis Group, 2025; Shen et al., 2024) |  |
| Global/Regional Estimates V5.GL.05 | WashU | 1998 - 2023 Annual & Monthly | x |  | x | x |  |  |  |  |  | x | x | x | x | x |  |  |  | x |  | x | x | x | x | x | (Atmospheric Composition Analysis Group, 2025; Hammer et al., 2023; Van Donkelaar et al., 2021) |  |
| North American Regional Estimates (V5.NA.04) | WashU | 2000 - 2022 Annual & Monthly | x |  |  |  |  |  |  |  |  | x | x | x | x | x |  |  |  | x |  |  |  |  |  | x | (Atmospheric Composition Analysis Group, 2025; Van Donkelaar et al., 2024) |  |
| v1 Empirical Models | CACES | 1999 - 2020† Annual |  |  |  | x | x | x | x | x | x | x | x | x | x | x |  |  |  | x |  |  |  |  | x |  | (CACES, 2025; Kim et al., 2020) |  |
| Spatially Decomposed v1 Empirical Models | CACES | 2000 - 2015 Annual |  |  |  |  |  |  |  |  | x | x | x | x | x | x |  |  |  | x |  |  |  |  | x |  | (Wang, 2020) |  |
| PM_2.5_ Concentrations for the CONUS | SEDAC | 2000 - 2016 Annual & Daily |  | x |  |  |  |  |  |  |  | x |  | x |  |  |  |  |  | x |  |  |  |  |  | x | (Di et al., 2019) |  |
| CalEnviro PM_2.5_ | OEHHA | Averaged between 2015 - 2017 |  |  |  |  |  |  |  | x |  |  |  | x |  |  |  |  |  | x |  |  |  |  |  |  | (OEHHA, 2015) |  |
| PM_2.5_ California | SJSU | 2006 - 2017 Daily |  |  | x |  |  |  |  |  |  |  | x |  |  |  |  |  |  | x | x |  |  |  |  |  | (Frank R. Freedman, 2017) |  |
| PM_2.5_ California Daily | SJSU | 7/10/19 - 8/10/21 Daily |  |  | x |  |  |  |  |  |  |  | x |  |  |  |  |  |  | x | x |  |  |  |  |  | (Frank R. Freedman, 2017) |  |

Table S2. Percentiles used to determine which percentile of satellite-derived PM_2.5_ values provide the strongest agreement with Environmental Protection Agency (EPA) monitor-based county-level design value (CDV) data. All percentiles from the 0.01 to the 0.99 were tested for multi-year periods (2016–2018 through 2021–2023) for Washington University in St. Louis (WashU) Global (GL) and North American (NA) datasets, calculating the Pearson and Spearman correlations between EPA CDVs and WashU GL and NA county-level design value equivalents (CDVEs).

| **WashU Global** | | | | |
| --- | --- | --- | --- | --- |
| Time Period | Best Pearson Percentile | Best Pearson r | Best Spearman Percentile | Best Spearman r |
| 2016-2018 | 0.98 | 0.815 | 0.97 | 0.778 |
| 2017-2019 | 0.98 | 0.801 | 0.94 | 0.780 |
| 2018-2020 | 0.85 | 0.759 | 0.95 | 0.755 |
| 2019-2021 | 0.75 | 0.735 | 0.93 | 0.726 |
| 2020-2022 | 0.78 | 0.751 | 0.94 | 0.724 |
| 2021-2023 | 0.95 | 0.741 | 0.98 | 0.705 |
|  | | | | |
| Best Overall Pearson Percentile | 0.882 |  | | |
| Best Overall Spearman Percentile | 0.952 |  | | |
| Best Percentile | 0.917 |  | | |
|  | | | | |
| **WashU North American** | | | | |
| Time Period | Best Pearson Percentile | Best Pearson r | Best Spearman Percentile | Best Spearman r |
| 2016-2018 | 0.96 | 0.770 | 0.98 | 0.752 |
| 2017-2019 | 0.96 | 0.762 | 0.97 | 0.747 |
| 2018-2020 | 0.76 | 0.706 | 0.94 | 0.748 |
| 2019-2021 | 0.81 | 0.694 | 0.96 | 0.725 |
| 2020-2022 | 0.82 | 0.701 | 0.93 | 0.718 |
|  | | | | |
| Best Overall Pearson Percentile | 0.862 |  | | |
| Best Overall Spearman Percentile | 0.956 |  | | |
| Best Percentile | 0.909 |  | | |


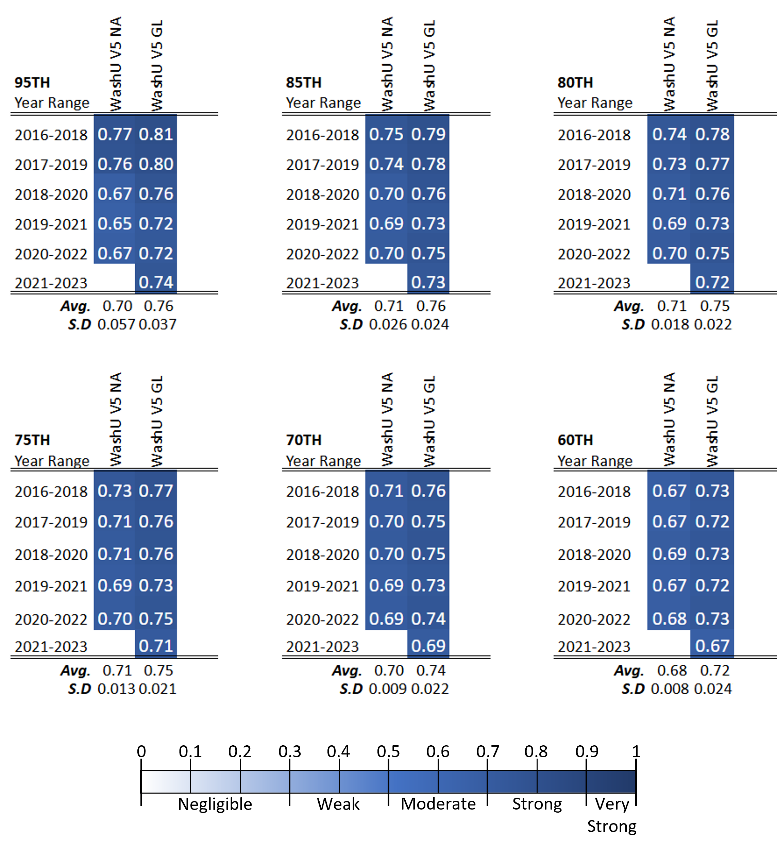


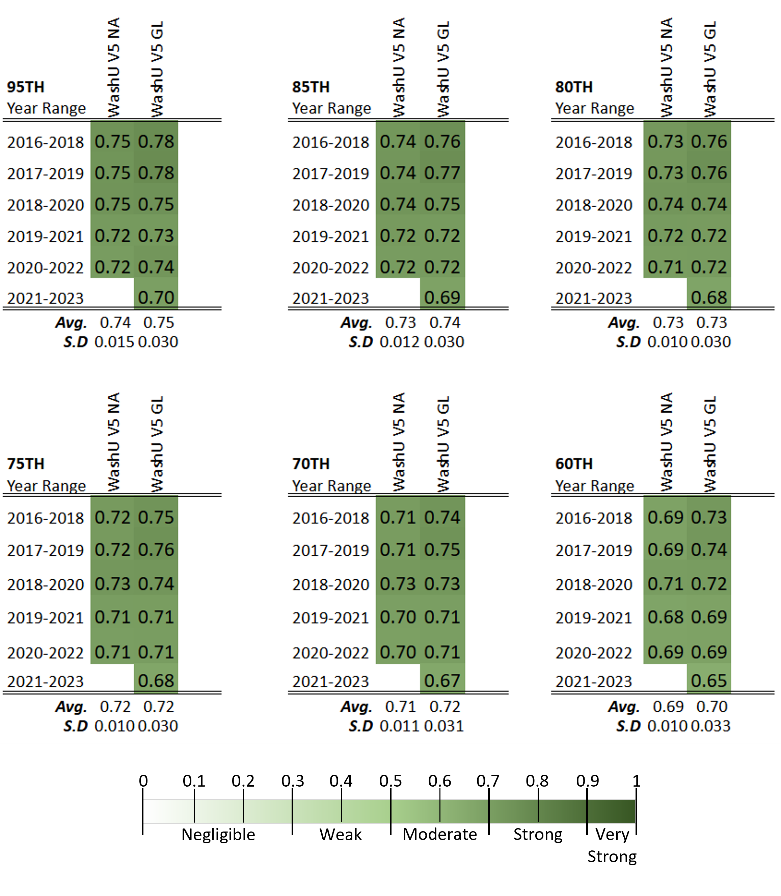


Figure S1. a) (Top) The 2016–2023 Pearson correlations, shown above in blue, for the WashU NA and WashU GL datasets using additional percentile methods: 95th, 85th, 80th, 75th, 70th, and 60th percentiles; b) (Bottom) The 2016–2023 Spearman rank correlations, shown below in green, for the WashU NA and WashU GL datasets using the same percentile methods.

**SI References**

Atmospheric Composition Analysis Group. (2025). *SatPM2.5 (Satellite-derived PM2.5) | Atmospheric Composition Analysis Group | Washington University in St. Louis*. https://sites.wustl.edu/acag/datasets/surface-pm2-5/

CACES. (2025). *CACES RCM/LUR Data Download*. CACES. https://www.caces.us/data

Di, Q., Amini, H., Shi, L., Kloog, I., Silvern, R., Kelly, J., Sabath, M. B., Choirat, C., Koutrakis, P., Lyapustin, A., Wang, Y., Mickley, L. J., & Schwartz, J. (2019). An ensemble-based model of PM2.5 concentration across the contiguous United States with high spatiotemporal resolution. *Environment International*, *130*, 104909. https://doi.org/10.1016/j.envint.2019.104909

Frank R. Freedman. (2017). *A SATELLITE-DISPERSION MODELING SYSTEM TO GENERATE HIGH-RESOLUTION DOWNSCALED PM2.5 FIELDS*. http://www.met.sjsu.edu/weather/HAQAST/articles/Freedman_CMAS2017_Technical_Abstract.pdf

Hammer, M. S., Van Donkelaar, A., Bindle, L., Sayer, A. M., Lee, J., Hsu, N. C., Levy, R. C., Sawyer, V., Garay, M. J., Kalashnikova, O. V., Kahn, R. A., Lyapustin, A., & Martin, R. V. (2023). Assessment of the impact of discontinuity in satellite instruments and retrievals on global PM2.5 estimates. *Remote Sensing of Environment*, *294*, 113624. https://doi.org/10.1016/j.rse.2023.113624

Kim, S.-Y., Bechle, M., Hankey, S., Sheppard, L., Szpiro, A. A., & Marshall, J. D. (2020). Concentrations of criteria pollutants in the contiguous U.S., 1979 – 2015: Role of prediction model parsimony in integrated empirical geographic regression. *PLOS ONE*, *15*(2), e0228535. https://doi.org/10.1371/journal.pone.0228535

OEHHA. (2015, April 30). *CAlEnviro Screen Download Data* [Text]. https://oehha.ca.gov/calenviroscreen/download-data

Shen, S., Li, C., van Donkelaar, A., Jacobs, N., Wang, C., & Martin, R. V. (2024). Enhancing Global Estimation of Fine Particulate Matter Concentrations by Including Geophysical a Priori Information in Deep Learning. *ACS ES&T Air*, *1*(5), 332–345. https://doi.org/10.1021/acsestair.3c00054

Van Donkelaar, A., Hammer, M. S., Bindle, L., Brauer, M., Brook, J. R., Garay, M. J., Hsu, N. C., Kalashnikova, O. V., Kahn, R. A., Lee, C., Levy, R. C., Lyapustin, A., Sayer, A. M., & Martin, R. V. (2021). Monthly Global Estimates of Fine Particulate Matter and Their Uncertainty. *Environmental Science & Technology*, *55*(22), 15287–15300. https://doi.org/10.1021/acs.est.1c05309

Van Donkelaar, A., Martin, R. V., Ford, B., Li, C., Pappin, A. J., Shen, S., & Zhang, D. (2024). North American Fine Particulate Matter Chemical Composition for 2000–2022 from Satellites, Models, and Monitors: The Changing Contribution of Wildfires. *ACS ES&T Air*, *1*(12), 1589–1600. https://doi.org/10.1021/acsestair.4c00151

Wang, Y. (2020). *Spatial decomposed concentrations of NO2 and PM2.5 air pollution in the United States at Census block group level* [Dataset]. Mendeley. https://doi.org/10.17632/CKDVX3D3ZC.1
